# Aberrant upregulation of 14-3-3ơ expression serves as an inferior prognostic biomarker for gastric cancer

**DOI:** 10.1186/1471-2407-11-397

**Published:** 2011-09-20

**Authors:** Wei-hua Zhou, Fang Tang, Jie Xu, Xing Wu, Zhi-ying Feng, Hai-gang Li, Dong-jun Lin, Chun-kui Shao, Quentin Liu

**Affiliations:** 1 Department of Hematology, the Third Affiliated Hospital, Sun Yat-Sen University, Guangzhou, China; 2 Department of Pathology, the Third Affiliated Hospital, Sun Yat-Sen University, Guangzhou, China; 3 State Key Laboratory of Oncology in South China, Cancer Center, Sun Yat-Sen University, Guangzhou, China; 4 Department of Pathology, Sun Yat-Sen Memorial Hospital, Sun Yat-Sen University, Guangzhou, China

## Abstract

**Background:**

14-3-3ơ is an intracellular, phosphoserine binding protein and proposed to be involved in tumorigenesis. However, the expression dynamics of 14-3-3ơ and its clinicopathological/prognostic significance in human tumors are still controversial.

**Methods:**

The method of immunohistochemistry (IHC) and Western blot were utilized to examine the protein expression of 14-3-3ơ in gastric cancer and paired normal adjacent gastric mucosal tissues. Receive operating characteristic (ROC) curve analysis was employed to determine a cutoff score for 14-3-3ơ expression in a training set (n = 66). For validation, the ROC-derived cutoff score was subjected to analysis of the association of 14-3-3ơ expression with patient outcome and clinical characteristics in a testing set (n = 86) and overall patients (n = 152).

**Results:**

The expression frequency and expression levels of 14-3-3ơ were significantly higher in gastric cancer than in normal gastric mucosal tissues. Correlation analysis demonstrated that high expression of 14-3-3ơ in gastric cancer was significantly correlated with clinical stage and tumor invasion. Furthermore, in the testing set and overall patients, Kaplan-Meier analysis showed that elevated 14-3-3ơ expression predicted poorer overall survival (OS) and progression-free survival (PFS). Importantly, high 14-3-3ơ expression was also associated with shortened survival time in stage III and stage IV gastric cancer patients. Multivariate analyses revealed that 14-3-3ơ expression was an independent prognostic parameter in gastric cancer.

**Conclusions:**

These findings provide evidence that high expression of 14-3-3ơ may be important in the tumor progression and servers as an independent molecular marker for poor prognosis of gastric cancer. Thus, overexpression of 14-3-3ơ identifies patients at high risk and is a novel therapeutic molecular target for this tumor.

## Background

Gastric cancer is one of the most common causes of cancer-related death worldwide [1], especially in East Asian countries such as China, Japan [2]. In 2005, there were approximately 0.4 million new cases and 0.3 million deaths from gastric cancer in China [3]. Despite recent advances in surgical techniques and medical treatment, the overall 5-year survival rate of gastric cancer in China remains low at about 40%. Many studies have demonstrated that multiple genetic alterations, including tumor suppressor genes, oncogenes, cell adhesion molecules, cell-cycle regulators, and growth factors, are responsible for the development and progression of gastric cancer [4]. Studies have unraveled many aberrantly expressed genes in gastric cancer including *BMI1 *[5], *COX-2 *[6], *HER3 *[7], *RKIP *and *STAT3 *[8], *SPARC *[9] and *HER2 *[10,11], which make risk assessment of gastric cancer patients more accurately. However, promising molecules that have clinicopathological/prognostic significance in gastric cancer remain substantially limited. It is necessary to further understand the molecular mechanisms involved in gastric cancer and to identify more valuable prognostic markers so as to not only improve poorer prognosis but also provide novel promising therapy targets.

14-3-3 proteins are a family of about 30 kD dimeric highly-conserved proteins and ubiquitously expressed in all eukaryotic organisms [12]. There are seven closely related genes (β, γ, ε, η, ζ, ơ,τ) in 14-3-3 family proteins. These genes often form heterodimers or homodimers and bind to over 100 different protein ligands [13,14]. Thus, 14-3-3 proteins are involved in many different cellular signaling processes including cell proliferation, cell cycle control, apoptosis and malignant transformation [14,15]. Among the seven isotypes, 14-3-3ơ was originally characterized as a human mammary epithelium marker 1 (HME1) and identified as a tumor suppress gene [12,16]. Growing evidences showed that 14-3-3ơ was significantly decreased or lost due to silencing of the gene via hypermethylation in several solid tumors [17-22]. Previous reports found that decreased expression of 14-3-3ơ predicted a poor survival in breast and nasopharyngeal cancer [23,24]. However, contradictory with the tumor suppressor role of 14-3-3σ, overexpression of 14-3-3ơ in pancreatic cancer cell led to drug resistance, cell migration and invasion [25]. Furthermore, high expression of 14-3-3σ was an independent prognostic factor for poor survival in pancreatic and colorectal cancer [26,27]. Thus, the biological role of 14-3-3ơ in tumorigenesis and progression of various types of human cancers varies depending on specific tumor type. Here, we selected the gastric cancer specimens with strict protocol to detect 14-3-3ơ expression dynamics and analyze their clinicopathological/prognostic significances.

## Methods

### Patients

A total of 216 primary gastric cancer patients from the archives of the Department of Pathology in the Third and Second Affiliated Hospital of Sun Yat-Sen University (Guangzhou, China) were initially recruited in our study. All patients underwent initial surgical resection from March 2001 to January 2006. We further screened patients using a strict eligibility criteria protocol as follows: microscopically confirmed adenocarcinoma of the stomach; without any metastatic diseases; no prior chemotherapy or radiation therapy history; receiving unified regimen as first-line chemotherapy after resection of primary tumors (we unified as FOLFOX regimen: fluorouracil, leucovorin and oxaliplatin), and no radiation treatment being administered to any of the patients; having over 5-year follow up period. Ultimately, 52 patients with loss of follow-up and 12 patients with deficiency in clinical characteristics were excluded from this study, leading to 152 gastric cancer patients subjecting to further clinical and survival analysis. In details, the overall cohort consisted of 105 male and 47 female with the median age of 58.0 year (range, 27 - 81 year). 103 patients were censused as death during the 5 years of follow-up time, including 5 cases died from postoperative complications and 98 cases died from tumor progression. Of the overall patients, 66 patients were randomly assigned by computer (SPSS 17.0 software) to the training set, and remaining 86 patients were randomly assigned to the testing set. Clinicopathological variables of the two cohorts, such as age, gender, clinical stage, tumor invasion, node stage, and histology differentiation, were included in this study. All tumors were classified and staged according to the revised guidelines advocated by the International Union against Cancer. We obtained prior patients' consent and approval from the Institute Research Ethics Committee of Sun Yat-Sen University for the use of clinical materials described in the present study.

### Tissue microarray construction

The tissue microarrays (TMAs) were constructed as a method described previously by Xie et al [28]. Briefly, the paraffin-embedded tissue blocks and the corresponding histological hematoxylin and eosin-stained slides were overlaid for TMA sampling. Interested core tissue biopsies (0.6-mm in diameter) were punched from representative tumor areas and from adjacent gastric mucosa tissue from blocks of individual donor tissue using a trephine (triplicate cylinders from carcinoma tissue and one cylinder from normal adjacent gastric mucosa tissue). The tissue cylinders were then transferred into a recipient paraffin block at defined positions by using a tissue-arraying instrument (Beecher Instruments, Silver Spring, MD, USA).

### Immunohistochemical analysis and evaluation

The TMAs slides were deparaffinized in xylene, rehydrated through graded alcohol, immersed in 3% hydrogen peroxide for 10 min to block endogenous peroxidase activity, and antigen retrieved by pressure cooking for 3 min in Tris/EDTA (pH = 8.0). Then the slides were incubated with the primary antibody of 14-3-3σ (monoclonal mouse; 1:50; Santa Cruz, SC-100638) for 1 hour at room temperature. After being incubated with the secondary antibody for 30 min, specimens were stained with DAB (3, 3-diaminobenzidine). Finally, the sections were counterstained with hematoxylin, dehydrated and mounted. A negative control was obtained by replacing the primary antibody with a normal murine IgG. Known immunostaining-positive colorectal carcinoma slides were used as positive controls as previously reported [27].

The brown granules in cytoplasm of 14-3-3σ were considered as positive staining. We scored the staining intensity as follows: 0, no staining; 1+, mild staining; 2+, moderate staining; 3+, intense staining. The area of staining was evaluated as follows: 0, no staining of cells in any microscopic fields; 1+, < 30% of tissue stained positive; 2+, between 30% and 60% stained positive; 3+, > 60% stained positive. 14-3-3σ expression was evaluated by combined assessing of staining intensity and extension. The minimum score when summed (intensity + extension) was 0, and the maximum was 6. The criteria used in this study has been widely accepted previously [24]. Expression of 14-3-3σ was assessed and scored by two independent pathologists (Drs. F Tang and ZY Feng) who were blinded to the clinicopathological data. The agreement of these two pathologists on the IHC score reached to 84% (128 identical scores in total 152 cases), suggesting our scoring system was highly solid and reproducible. If the results reported by the two pathologists were consistent, the value was selected. However, the interobserver disagreements (approximately 6% of total cases) were reviewed for a second time, followed by a conclusive judgment by both pathologists.

### Western blot analysis

The gastric cancer and paired normal adjacent gastric mucosal tissues were ground and lysed with the RIPA buffer on ice before being subjected to Western blot analysis. The protein concentration was detected by the Bradford method with BSA (Sigma-Aldrich) as the standard. Equal amounts of cell and tissue extract (40 μg) were subjected to SDS-PAGE and transferred to nitrocellulose membrane (Bio-Rad) for antibody blotting. The membrane was then blocked and incubated with mouse anti-glyceraldehyde 3-phosphate dehydrogenase (GAPDH) antibody (Abmart, #M20006) and mouse anti-14-3-3σ antibody (Santa Cruz, SC-100638).

### Selection of cutoff score for 14-3-3σ "positive" expression

The ROC curve analysis was subjected to the selection of 14-3-3σ cutoff score in the training set, as described previously [29]. Briefly, the sensitivity and specificity for the outcome being studied at each score was plotted to generate a ROC curve. The score localized closest to the point at both maximum sensitivity and specificity, the point (0.0, 1.0) on the curve, was selected as the cutoff score leading to the greatest number of tumors which were correctly classified as having or not having the outcome. To facilitate ROC curve analysis, the survival features were dichotomized: survival (death VS. others (censored, alive or death from other causes)).

### Follow up

All patients had follow-up records for over 5 years. After the completion of therapy, patients were observed at 3 month intervals during the first 3 years and at 6 month intervals thereafter. Overall survival was defined as the time from diagnosis to the date of death or when censused at the latest date if patients were still alive. Progression-free survival was defined as the time from diagnosis to the date of local failure/distant metastasis or the date of death or when censused at the latest date.

### Statistical analysis

For survival analysis, optimal cutpoint for 14-3-3σ expression was obtained by ROC analysis in the training set (n = 66). For validation, the relationships between 14-3-3σ expression, which was classified by ROC analysis-generated cutoff point, and OS, PFS were evaluated in the testing set (n = 86) and overall patients (n = 152). The chi-square test or Fisher's exact test was employed to evaluate the relationship between 14-3-3σ expression and clinicopathological variables. The multivariate Cox proportional hazards model was utilized to estimate the hazard ratios and 95% confidence intervals for patient outcome. The relationships between 14-3-3σ expression and OS, PFS were determined by Kaplan-Meier analysis. The log-rank tests were performed to value the difference in survival probabilities between patient subsets. All *p *values quoted were two-sided and *p *< 0.05 was considered statistically significant. Statistical analysis was performed using SPSS v. 17.0 (SPSS, Inc, Chicago, IL).

## Results

### 14-3-3σ expression in gastric cancer and normal gastric mucosal tissues

**Figure 1:**
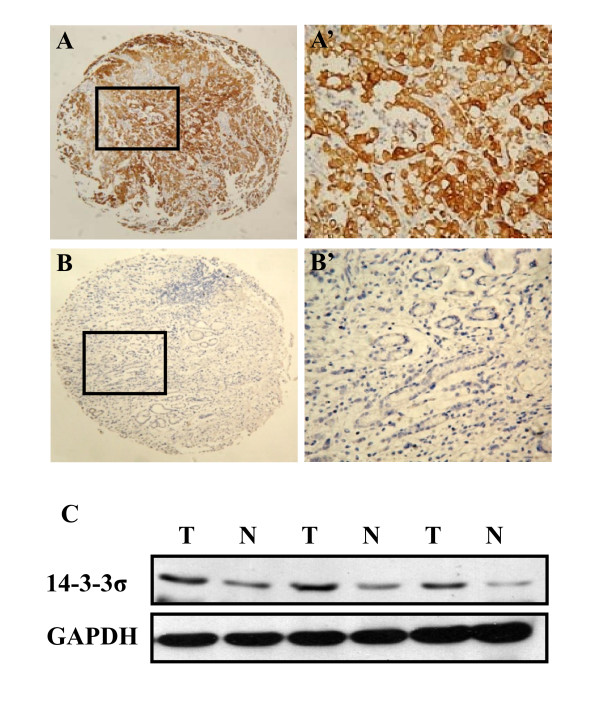
**14-3-3σ expression in human primary gastric cancer and normal adjacent gastric mucosal tissue**. (A) 14-3-3σ was detected in the cytoplasm and overexpressed in the gastric cancer tissue (100 ×). (B) Adjacent non-neoplastic gastric mucosal tissue showed nearly negative expression of 14-3-3σ (100 ×). (A'), (B') demonstrated the higher magnification (400 ×) from the area of the box in (A) and (B) respectively. (C) Western blot analysis of 14-3-3σ expression in representative primary gastric cancer tissue (T) and normal adjacent mucosal tissue (N). Equal loading of protein was determined by GAPDH.

14-3-3σ was detected in the cytoplasm and overexpressed in the gastric cancer (Figure 1A and 1A'), whereas normal paired gastric mucosal tissues showed nearly negative expression (Figure 1B and 1B'). Consistent with this result, Western blot analysis revealed a similar finding in gastric cancer and normal adjacent gastric mucosal tissues (Figure 1C).

**Figure 2:**
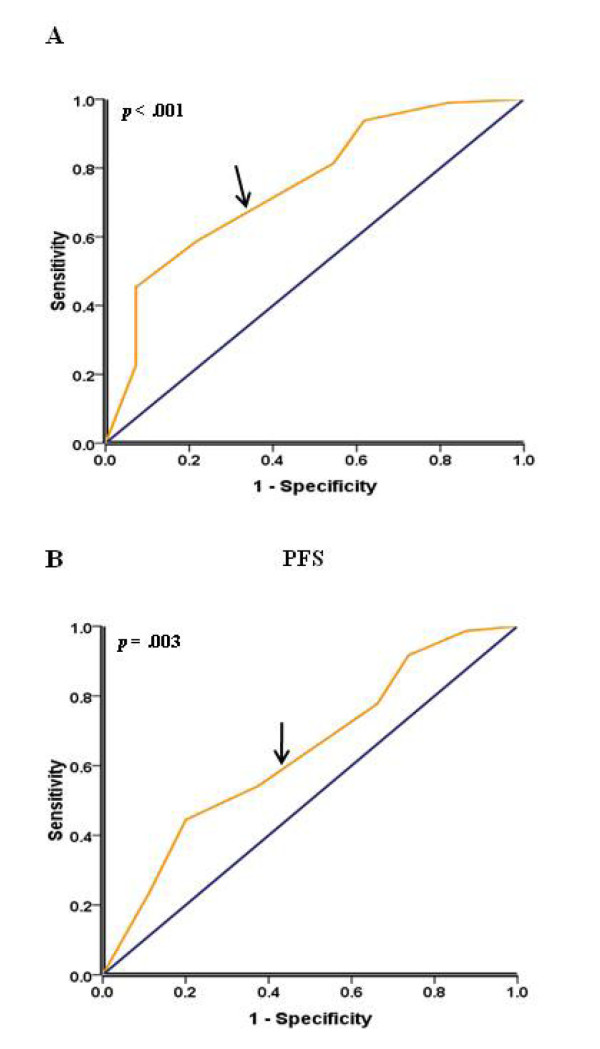
**Receiver operating characteristic (ROC) curves analysis of 14-3-3σ cutoff score in the training set**. (A) 14-3-3σ cutoff point for overall survival in the training set. (B) 14-3-3σ cutoff point for progression-free survival in the training set. At each immunohistochemical score, the sensitivity and specificity for the outcome being studied was plotted, thus generating a ROC curve. 14-3-3σ cutoff score for overall survival, progression-free survival was 3.25 and 3.35 respectively.

To further assess survival analysis and avoid the problems of multiple cutpoint selection, ROC curve analysis was employed to determine cutoff score for 14-3-3σ expression. As shown in Figure 2A and 2B, the 14-3-3σ cutoff score for OS and PFS in the training set was 3.25 (*p *< 0.001) and 3.35 (*p *= 0.003) respectively. We thus selected a 14-3-3σ expression score of 3 (> 3 VS. ≤ 3) as the uniform cutoff point for survival analysis in the testing set.

### 14-3-3σ expression and clinical features

The clinical features of these two cohorts of patients, including age, gender, clinical stage, tumor invasion, node stage, histology differentiation, 14-3-3σ expression, were summarized in Table 1. ROC-derived 14-3-3σ cutoff score of 3 in the training set successfully segregated the testing set into high (44/86, 51.2%) and low (42/86, 48.8%) 14-3-3σ expression subgroups. High expression of 14-3-3σ was mainly found in more advanced tumor stages (77/107 in stage III+IV VS. 12/45 in stage I+II, *p *= 0.004). Furthermore, correlation analysis demonstrated that high 14-3-3σ expression was correlated with clinical stage (*p *< 0.001 for both set) and tumor invasion (*p *< 0.001 for training set and *p *= 0.005 for testing set) in both sets. 14-3-3σ associated to patients' age in the testing set (*p *= 0.01) but not in the training set. We failed to detect any relationship between 14-3-3σ with other patient characteristics, including gender, node stage, and histology differentiation.

**Table 1:** Association of 14-3-3σ expression with patient's characteristics in primary gastric cancer

Variable	All cases	Training set (n = 66)			Testing set (n = 86)		
		
		High expression	Low expression	*p ^a^*	High expression	Low expression	*p ^a^*
**Age (years)**							
≥ 58.00 ^b ^	83	25(30.1%)	13(15.7%)	0.627	29(34.9%)	16(19.3%)	0.010
< 58.00	69	20(29.0%)	8(11.6%)		15(21.7%)	26(37.7%)	
**Gender**							
Male	105	29(27.6%)	16(15.2%)	0.340	31(29.5%)	29(27.6%)	0.887
Female	47	16(34.0%)	5(10.6%)		13(27.7%)	13(27.7%)	
**Clinical stage**							
I + II	45	6(13.3%)	12(26.7%)	0.000	6(13.3%)	21(46.7%)	0.000
III + IV	107	39(36.4%)	9(8.4%)		38(35.5%)	21(19.6%)	
**Tumor invasion**							
T_1_+T_2_	23	0(0%)	7(30.4%)	0.000	3(13.0%)	13(56.5%)	0.005
T_3_+T_4_	129	45(34.9%)	14(10.9%)		41(31.8%)	29(22.5%)	
**Node stage**							
N_0_+N_1_	101	32(31.7%)	15(14.9%)	0.979	24(23.8%)	30(29.7%)	0.105
N_2_+N_3_	51	13(25.5%)	6(11.8%)		20(39.2%)	12(23.5%)	
**Histology-differentiation**							
Well	51	15(29.4%)	11(21.6%)	0.123	11(21.6%)	14(27.5%)	0.395
Poorly	101	31(30.7%)	10(9.9%)		33(32.7%)	28(27.7%)	

^a ^x^2 ^test

^b ^median age

### 14-3-3σ expression and survival analysis: univariate survival analysis

**Figure 3:**
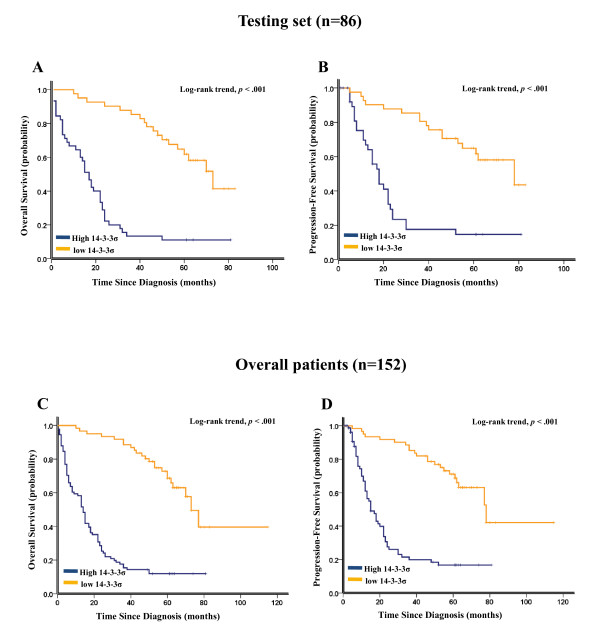
**Kaplan-Meier survival analysis of 14-3-3σ expression in the testing set and overall patients**. (A) Higher 14-3-3σ expression was closely correlated with poor overall survival and (B) progression-free survival in the testing set. (C) Patients with higher 14-3-3σ expression also acquired an inferior overall survival and (D) progression-free survival in overall patients. In the testing set and overall patients, the median duration of overall survival for patients with low and high expression of 14-3-3σ was 73.0 VS. 17.0 months (*p *< 0.001) and 73.0 VS. 14.0 months (*p *< 0.001), respectively.

**Figure 4:**
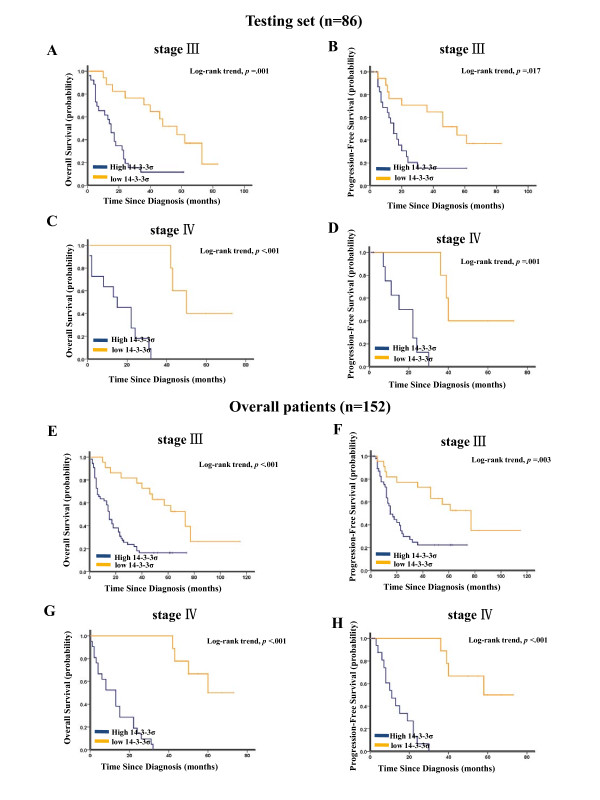
**Kaplan-Meier survival analysis of 14-3-3σ expression in subsets of gastric cancer patients with stage III and IV (log-rank test)**. (A) Probability of overall survival and (B) progression-free survival of patients with stage III gastric cancer in the testing set: low expression, n = 17; high expression, n = 26; (C) Probability of overall survival and (D) progression-free survival of stage IV patients with gastric cancer in the testing set: low expression, n = 5; high expression, n = 11. (E) Probability of overall survival and (F) progression-free survival of patients with stage III gastric cancer in the overall patients: low expression, n = 22; high expression, n = 55; (G) Probability of overall survival and (H) progression-free survival of stage IV patients with gastric cancer in the overall patients: low expression, n = 9; high expression, n = 21.

As shown in Figure 3A and 3C, Kaplan-Meier analysis showed that elevated 14-3-3σ expression strongly predicted an inferior OS in the testing set (*p *< 0.001) and overall patients (*p *< 0.001). Moreover, 14-3-3σ expression was also a powerful prognostic factor for PFS in the testing set (*p *< 0.001, Figure 3B) and overall patients (*p *< 0.001, Figure 3D). Considering the poor outcome in late-stage gastric cancer, further analysis was performed with regard to 14-3-3σ expression in subsets of gastric cancer patients with stage III and IV. The results demonstrated that compared with 14-3-3σ low expression, high expression of 14-3-3σ showed a significant trend toward worse OS and PFS in gastric cancer patients of stage III (*p *= 0.001 for OS and *p *= 0.017 for PFS, Figure 4A and 4B) and stage IV (*p *< 0.001 for OS and *p *= 0.001 for PFS, Figure 4C and 4D). Results in the overall patients were similar to those found in the testing set (Figure 4E-H).

### Multivariate Cox regression analysis

To avoid the influence caused by univariate analysis, the expression of 14-3-3σ as well as other parameters was examined in multivariate Cox analysis (Table 2 and Table 3). In the testing set, 14-3-3σ was indeed found to be a significant independent prognostic factor for poor OS (hazard ratio, 4.527; 95% CI, 2.274-9.012; *p *< 0.001; Table 2) and PFS (hazard ratio, 3.582; 95% CI, 1.717-7.475; *p *= 0.001; Table 2). Similar results were also observed in overall patients (hazard ratio, 5.161; 95% CI, 2.990-8.909; *p *< 0.001 for OS and hazard ratio, 4.416; 95% CI, 2.482-7.856; *p *< 0.001 for PFS; Table 3). Of other parameters, clinical stage was found to be an independent prognostic factor for patient survival in the testing set but not in overall patients.

**Table 2:** Results of multivariate Cox proportional-hazards analysis in testing set

Variable	For death			For progression-free survival		
	
	Hazard Ratio	95% confidence interval	*p*	Hazard Ratio	95% confidence interval	*p*
**Age < 58.00** **years (VS. ≥ 58 years)**	1.101	0.637 to 1.902	0.731	1.056	0.577 to 1.936	0.859
**Gender Male** **(VS. Female)**	0.772	0.408 to 1.461	0.427	1.049	0.525 to 2.094	0.892
**Clinical stage** **IV + III (VS. II +I)**	3.391	1.156 to 9.949	0.026	5.220	1.529 to 17.823	0.008
**Tumor invasion** **T_4 _+ T_3 _(VS. T_2 _+ T_1_)**	1.066	0.320 to 3.553	0.917	0.730	0.206 to 2.585	0.626
**Nodal stage N_3 _+ N_2_** **(VS. N_1 _+ N_0_)**	0.705	0.387 to 1.282	0.251	0.735	0.373 to 1.448	0.373
**Differentiation** **Low (VS. High)**	0.862	0.437 to 1.701	0.668	0.936	0.442 to 1.982	0.863
**14-3-3σ** **Positive (VS. Negative)**	4.527	2.274 to 9.012	0.000	3.582	1.717 to 7.475	0.001

**Table 3:** Results of multivariate Cox proportional-hazards analysis in overall patients

Variable	For death			For progression-free survival		
	
	Hazard Ratio	95% confidence interval	*p*	Hazard Ratio	95% confidence interval	*p*
**Age < 58.00** **years (VS. ≥ 58 years)**	1.046	0.701 to 1.562	0.825	1.036	0654 to 1.642	0.880
**Gender Male** **(VS. Female)**	0.713	0.474 to1.073	0.105	0.733	0.409 to 1.278	0.165
**Clinical stage** **IV + III (VS. II +I)**	0.988	0.500 to1.952	0.972	1.355	0.558 to 3.121	0.476
**Tumor invasion** **T_4 _+ T_3 _(VS. T_2 _+ T_1_)**	2.175	0.896 to 5.278	0.086	1.432	0.547 to 3.747	0.465
**Nodal stage N_3 _+ N_2_** **(VS. N_1 _+ N_0_)**	1.056	0.681 to1.638	0.808	1.243	0.751 to 2.059	0.397
**Differentiation** **Low (VS. High)**	0.765	0.470 to1.243	0.279	0.810	0.478 to1.375	0.463
**14-3-3σ** **Positive (VS. Negative)**	5.161	2.990 to 8.909	0.000	4.416	2.482 to 7.856	0.000

## Discussion

Gastric cancer is one of the most common cancers worldwide and it poses one of the most serious public health problem [1]. Recurrence and metastasis are still the major issues for the poor survival of advanced gastric cancer patients [30]. Although previous studies have found that many aberrant expressed genes in gastric tumor can help classify the risk of patient outcome [5-11], more novel molecular markers that can identify tumor progression and predict the prognosis individually are still urgently needed. The 14-3-3 family proteins have gained much attention over the past years due to their involvement in cancers by regulation of diverse cellular processes [14,15]. Among the seven isoforms, 14-3-3σ is up-regulated by p53 in response to DNA damage, and sequesters the essential mitotic initiation complex, cdc2-cyclin B1, from entering the nucleus, thus preventing the initiation of mitosis [31]. As a result, 14-3-3σ induces G2 arrest and allows DNA damage repair. Thus, different from other members of the family, 14-3-3σ is defined as a negative regulator of cell cycle checkpoints and a potential tumor-suppressor protein [12]. However, the biological role of 14-3-3ơ in tumorigenesis and progression of various types of human tumors remains controversial. Here, to further reveal the biological function of 14-3-3ơ in gastric cancer, we studied 14-3-3ơ expression dynamics and analyzed their clinicopathological/prognostic significances in 152 tumor specimens.

In this study, to develop an objective 14-3-3σ cutoff point for survival analysis, we used the ROC curve analysis to generate a cutoff score in the training set. 14-3-3σ expression, which was classified as high and low level by the ROC-derived cutoff point, was mainly found to be higher in more advanced tumor stages (stage III and IV), indicating that 14-3-3σ might be involved in gastric cancer progression. Correlation analysis further demonstrated that high 14-3-3σ expression was associated with clinical stage and tumor invasion in gastric cancer (Table 1). Furthermore, in the testing set and overall patients, high 14-3-3ơ expression predicted a significant OS and PFS disadvantage over low 14-3-3ơ expression subgroup (Figure 3). Importantly, worse prognostic impact of increased 14-3-3σ expression was demonstrated in patients with stage III and IV tumors (Figure 4), indicating that 14-3-3ơ might be a novel factor for risk definition in gastric cancer. In addition, multivariate analyses in the testing set and overall patients revealed that 14-3-3ơ expression was an independent prognostic parameter. Taken together, our findings in this study provided evidence that elevated expression of 14-3-3σ in gastric cancer might facilitate an increased malignant and worse prognostic phenotype of this tumor.

With regard to the prognostic impact of 14-3-3σ in different human cancers, some of the reported data are totally contradictory. It was documented that loss expression of 14-3-3σ was linked to a poor prognosis of patients with breast and nasopharyngeal cancers [23,24]. However, consistent with the results found in pancreatic and colorectal carcinomas [26,27], our finding in this study showed that high expression of 14-3-3σ was positively associated with clinic stage and poor survival. The underlying mechanism(s) of 14-3-3σ to impact cancer prognosis might be depended on intrinsic properties of the tumor type. Moreover, different co-expression of 14-3-3σ and other molecules [24], as well as the nature of the therapeutic regimen in various types of human cancers [32], which further make us to understand that the function of 14-3-3σ and its underlying mechanism(s) to impact cancer prognosis may be tumor-type specific. Recently, studies have showed that overexpression of 14-3-3σ is correlated with tumor metastasis and progression in pancreatic [25], breast [33] and ovarian cancer cells [34], which may further explain our findings that higher 14-3-3σ expression is mainly detected in more advanced tumor stages and also identified as a poor prognosis indicator in late-stage gastric cancer patients. Hence, our study demonstrated that 14-3-3σ was an independent prognostic biomarker for OS and PFS in gastric cancer. Especially, overexpression of 14-3-3σ was relevant with tumor progression. The findings reported here could have clinical value in predicting the prognosis of gastric cancer and identifying gastric cancer patients that are at high risk of progression and recurrence.

## Conclusion

In summary, in this study, we describe 14-3-3σ expression in gastric cancer and normal gastric mucosal tissues. Our results provide a basis for the concept that increased expression of 14-3-3σ in human gastric cancer may be important in the tumor progression and serves as an independent biomarker for poor survival. Thus, overexpression of 14-3-3ơ identifies patients at high risk and is a novel therapeutic molecular target for gastric cancer.

## Competing interests

The authors declare that they have no competing interests.

## Authors' contributions

WHZ designed the study, evaluated the clinical records and drafted the manuscript. FT carried out the immunohistochemistry assays and performed the immunohistochemical analyses. JX participated in the statistical analysis, carried out the Western blot assay and performed the immunohistochemical analyses. XW helped to draft the manuscript. ZYF performed the immunohistochemical analyses. HGL and DJL analyzed the data. CKS and QL participated in the design of the study, in its analysis and in the interpretation of the data. QL also participated in writing the manuscript. All authors read and approved the final manuscript.

## Pre-publication history

The pre-publication history for this paper can be accessed here:

http://www.biomedcentral.com/1471-2407/11/397/prepub
